# Microglia-Mediated Vascular Network Remodeling After Ischemic Stroke: An Immunovascular Repair Framework

**DOI:** 10.3390/cells15151322

**Published:** 2026-07-24

**Authors:** Xinyu Li, Xiang Li, Yushi Li, Yuping Kang, Liangqin Shi

**Affiliations:** 1 Chengdu University of Traditional Chinese Medicine, Chengdu 611137, China; 2 School of Medical and Life Sciences, Chengdu University of Traditional Chinese Medicine, Chengdu 611137, China; 3 School of Basic Medical Sciences, Chengdu University of Traditional Chinese Medicine, Chengdu 611137, China; 4 College of Medical Technology, Chengdu University of Traditional Chinese Medicine, Chengdu 611137, China

**Keywords:** repair-associated microglia, M2d-like features, ischemic stroke, vascular network remodeling, neurovascular unit, angiogenesis, blood–brain barrier, extracellular vesicles, traditional Chinese medicine

## Abstract

Ischemic stroke remains a leading cause of death and long-term disability worldwide. Although acute reperfusion therapies have improved outcomes in selected patients, effective strategies that directly promote neurovascular repair during the subacute and chronic phases remain limited. Vascular network remodeling in the peri-infarct region is increasingly recognized as a key process supporting tissue repair, blood–brain barrier restoration, and functional recovery after stroke. Microglia, as resident immune cells of the central nervous system, undergo dynamic morphological, metabolic, and functional changes after ischemic injury and participate in inflammation, phagocytic clearance, blood–brain barrier regulation, and tissue repair. Among repair-associated microglial states, microglia with M2d-like features have attracted increasing attention because of their potential association with immunoregulation and pro-vascular repair. However, whether repair-associated microglia with M2d-like features represent a distinct and stable microglial subtype after stroke remains unresolved. In this review, we summarize current evidence linking repair-associated microglial responses to vascular network remodeling after ischemic stroke, with particular emphasis on the conceptual value of the M2d-like state. We discuss putative mechanisms involving paracrine signaling, perivascular localization, metabolic reprogramming, and extracellular vesicle-mediated communication. We also evaluate therapeutic implications, including traditional Chinese medicine, extracellular vesicle-based strategies, and nanodelivery systems. However, current therapeutic evidence does not establish that these interventions specifically induce M2d-like microglial states. We highlight the need for rigorous validation of cellular identity, spatial localization, and functional vascular outcomes. Overall, the M2d-like framework provides a candidate perspective for understanding immune–vascular coupling after stroke, but further studies integrating single-cell omics, spatial mapping, lineage tracing, and functional vascular assessment are required to define the identity and functional contribution of repair-associated microglia with M2d-like features. Method: This article is a narrative review. The relevant literature was searched in PubMed from database inception to June 2026 using combinations of the terms “ischemic stroke,” “microglia,” “macrophage,” “vascular remodeling,” “angiogenesis,” “M2d,” “extracellular vesicles,” “traditional Chinese medicine,” and “nanomedicine.” Priority was given to original studies directly examining microglial or myeloid responses and vascular repair after ischemic stroke. Relevant review articles were included to provide conceptual background. Because direct evidence for M2d-like microglial responses after stroke remains limited, selected studies involving peripheral macrophages, tumor-associated macrophages, traditional Chinese medicine, extracellular vesicles, and nanomedicine were included as indirect or hypothesis-generating evidence. Evidence was interpreted according to the disease model, cellular source, and vascular outcomes examined, with stroke-specific microglial studies regarded as more directly relevant than evidence extrapolated from non-stroke or non-microglial models.

## 1. Introduction

### 1.1. Definition of Ischemic Stroke

Ischemic stroke accounts for approximately 70–80% of all strokes and remains a major cause of disability and death worldwide [1]. Although acute therapeutic strategies, including thrombolytic therapy and endovascular thrombectomy, have improved clinical outcomes in selected patients, only a limited proportion of patients are eligible for reperfusion therapy because of the narrow therapeutic window and strict clinical criteria [2,3]. Moreover, current clinically effective interventions remain largely focused on acute-phase reperfusion, whereas few strategies directly promote neurovascular repair and functional recovery during the subacute and chronic phases [4]. Therefore, promoting vascular remodeling and endogenous tissue repair after ischemia represents an important direction for improving post-stroke recovery.

### 1.2. Importance of Vascular Remodeling After Ischemic Stroke

**Figure 1:**
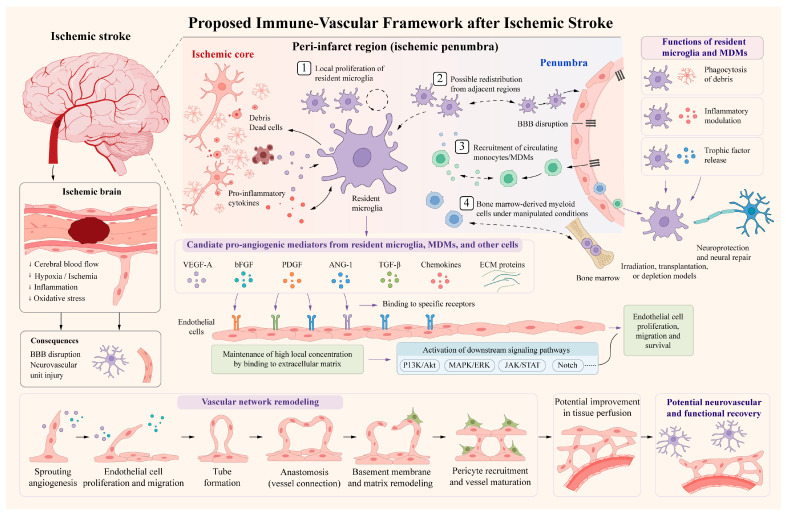
Microglial mechanisms regulating vascular network remodeling and neurovascular repair after ischemic stroke. Ischemic injury induces hypoperfusion, hypoxia, inflammation, and oxidative stress, causing blood–brain barrier (BBB) disruption and neurovascular unit damage. In response, post-stroke myeloid responses involve local proliferation of resident microglia, possible redistribution from adjacent regions, recruitment of circulating monocytes/monocyte-derived macrophages, and, under manipulated experimental conditions, entry of bone marrow-derived myeloid cells. Activated microglia execute debris phagocytosis, inflammatory regulation, and trophic factor release to promote neuroprotection. Concurrently, microglia and neighboring cells secrete pro-angiogenic factors (including VEGF-A, bFGF, PDGF, ANG-1, TGF-β, chemokines, and ECM proteins). These factors bind to specific endothelial receptors and matrix components to maintain local concentration gradients, activating downstream PI3K/Akt, MAPK/ERK, JAK/STAT, and Notch signaling pathways. This molecular activation drives endothelial cell proliferation, migration, and survival, initiating a sequential vascular remodeling cascade: sprouting angiogenesis, tube formation, anastomosis, basement membrane synthesis, and pericyte recruitment. This orchestrated process ultimately re-establishes functional blood flow and promotes neurological recovery. The figure was created using Adobe Illustrator 2024 (Adobe Inc., San Jose, CA, USA).

Cerebral ischemia and hypoxia initiate a cascade of pathophysiological events after ischemic stroke. Within this context, vascular regeneration is increasingly recognized as a key process in post-stroke repair. Clinical and experimental studies have shown that increased microvessel density in the peri-infarct region is associated with longer survival in patients with ischemic stroke [4,5]. Neovascularization after stroke may restore blood supply to ischemic tissue and support neurogenesis, thereby contributing to neurological recovery [6]. Accordingly, enhancing vascular network remodeling in ischemic regions is considered a promising strategy for promoting functional recovery after stroke. Vascular network remodeling involves the formation and expansion of new vascular networks from pre-existing capillaries or microvessels through sprouting and non-sprouting mechanisms [7,8]. This process includes protease production, endothelial cell proliferation and migration, tube formation, anastomosis between newly formed vascular tubes, basement membrane synthesis, and recruitment of pericytes and smooth muscle cells [9]. Vascular remodeling is regulated by multiple cell types and biomolecules, including vascular growth factors, chemokines, and extracellular matrix-associated proteins [10]. These factors are typically secreted by cells located near growing vessels, such as endothelial cells, macrophages, platelets, and microglia [11,12,13]. After release, they bind to endothelial receptors and extracellular matrix components, thereby maintaining local concentration gradients that create a microenvironment permissive for neovascularization [14]. These gradients activate downstream signaling pathways and support the formation of new vessels [15] (Figure 1). Thus, the establishment of spatially organized pro-angiogenic signals is critical for vascular network remodeling after ischemic stroke.

### 1.3. Role of Microglia in Ischemic Stroke

Microglia, the resident immune cells of the central nervous system (CNS), play a central role in the brain immune response to ischemic stroke [16]. After ischemic injury, microglia are rapidly activated and initiate a complex response that influences inflammation, tissue repair, and the maintenance of brain homeostasis [17]. Beyond acute injury responses, microglia contribute to long-term brain homeostasis by regulating synaptic plasticity and neural network stability [18,19]. They also mediate phagocytic clearance of cellular debris and dead neurons, which is essential for creating a microenvironment conducive to tissue repair [20].

Although the roles of microglia in post-stroke inflammation and tissue repair have been extensively investigated, the mechanisms by which microglial phenotypic transitions regulate the blood–brain barrier and contribute to vascular network remodeling remain incompletely understood. Because microglial functions change dynamically across injury stages and in response to local microenvironmental cues, elucidating their phenotypic plasticity may provide important insights into post-stroke vascular repair. This review therefore evaluates the extent to which current evidence supports an association between repair-associated microglial responses with M2d-like features and post-stroke vascular remodeling, while identifying key evidentiary gaps and future research priorities.

## 2. Microglia Activation After Ischemic Stroke

### 2.1. Morphological and Functional Changes

During the early stage of ischemic injury, microglia are among the first resident immune cells in the CNS to respond. Under homeostatic conditions, microglia exhibit a highly ramified morphology, with fine processes that continuously extend and retract to survey the surrounding brain parenchyma [16]. After stroke onset, microglia undergo rapid morphological remodeling, characterized by enlargement of the cell body, shortening of cellular processes, and reduced branching. They gradually shift from a ramified morphology to a deramified or amoeboid phenotype, indicating a transition from a homeostatic surveillance state to a reactive state [21]. These morphological and phenotypic changes mark the initiation of the brain response to ischemic injury and suggest that microglial functions begin to shift toward inflammatory regulation, migration, phagocytic clearance, and repair-associated responses.

Notably, microglial morphological changes are not static structural alterations but are dynamically regulated by local perfusion and energy status. In ischemic penumbra-like regions, reduced capillary blood flow around microglial somata may be accompanied by decreased process motility and the onset of deramification. By contrast, complete cessation of blood flow can rapidly halt process activity, although typical deramification may not occur immediately within a short time window [22]. Therefore, post-stroke morphological and functional changes in microglia should be interpreted as a continuous and dynamic process. The functional consequences of microglial activation are closely related to injury stage, local perfusion status, and the inflammatory microenvironment.

### 2.2. Resident Microglia and Infiltrating Myeloid Cells After Ischemic Stroke

Resident microglia originate from yolk sac-derived primitive myeloid progenitors during embryonic development. Fate-mapping studies further indicate that adult microglia predominantly derive from primitive myeloid progenitors established during early development, with minimal contribution from postnatal hematopoietic progenitors under homeostatic conditions [23,24]. Following central nervous system injury, reactive microgliosis is accompanied by local expansion and proliferation of resident microglia [25]. Experimental depletion studies also indicate that microglial repopulation can arise from central nervous system-resident cells rather than circulating bone marrow-derived precursors [26].

After ischemic stroke, peripheral monocytes and macrophages may infiltrate injured brain tissue and contribute to local inflammatory and repair-related responses [21]. Although these infiltrating cells can acquire morphological and molecular characteristics resembling those of activated microglia, they remain distinct from resident microglia in developmental origin and recruitment route. Therefore, resident microglia and infiltrating monocyte-derived macrophages should be distinguished when interpreting post-stroke myeloid responses.

Resident microglial identity is commonly supported by combinations of lineage-associated markers such as TMEM119, P2RY12, and SALL1, whereas Iba1, CD11b, CD68, and CX3CR1 are shared by multiple myeloid populations and cannot independently establish resident microglial identity [27]. Similarly, CD163 and CD206 may be expressed by activated microglia and other brain-associated macrophage populations. IL-10 and VEGF-A are characteristic components of the immunoregulatory and pro-angiogenic program described in M2d macrophages and are therefore treated here as functional rather than lineage-defining features [28]. Thus, cellular lineage identity should be evaluated independently of functional activation state. Molecules such as CD163, CD206, IL-10, and VEGF-A may reflect repair-associated or pro-vascular functions, but their expression alone is insufficient to establish that the responding cells are resident microglia (Table 1).

## 3. Microglial Polarization and Functional States After Ischemic Stroke

### 3.1. M1-like and M2-like Functional Programs After Ischemic Stroke

After ischemic stroke, microglia rapidly transition from a homeostatic surveillance state to reactive states involved in injury recognition, inflammatory regulation, cellular debris clearance, and tissue repair [31]. The M1-like and M2-like framework is commonly used as a simplified description of broad functional tendencies after ischemic injury [32]. M1-like responses are broadly associated with pro-inflammatory and potentially injury-amplifying activities, whereas sustained activation may compromise endothelial function and blood–brain barrier integrity [33]. In contrast, M2-like responses are more closely associated with inflammation resolution, phagocytic clearance, extracellular matrix regulation, and tissue repair [34]. During the post-stroke repair phase, these responses may indirectly support vascular remodeling by reducing inflammatory burden and improving the local reparative microenvironment [35]. Within the conventional macrophage polarization framework, M2-like responses have been further subdivided into M2a-like, M2b-like, M2c-like, and M2d-like programs [36]. Representative stimuli, markers, mediators, and proposed relationships with post-stroke vascular repair are summarized in Table 2. These subdivisions were largely established in macrophage studies and should therefore be applied cautiously to microglia in vivo. Among these programs, the M2d-like designation is commonly used to describe a pro-angiogenic and immunoregulatory myeloid response characterized by VEGF and IL-10 upregulation. However, whether a distinct and stable microglial population exhibiting this program exists after ischemic stroke remains unresolved [28].

Although the M1/M2 classification has been widely used to describe inflammatory and reparative microglial responses, this traditional framework does not fully capture the complexity of microglial activation [37]. High-throughput approaches, including single-cell transcriptomics, have shown that microglia can adopt diverse reactive states with distinct molecular signatures across developmental stages, brain regions, and disease contexts [38]. In pathological conditions in vivo, microglia may display mixed or transitional features and can simultaneously express markers associated with both M1-like and M2-like programs [39]. Microglial activation after stroke is therefore not confined to a single M1-M2 axis but involves multiple overlapping programs, including inflammatory responses, phagocytic clearance, antigen presentation, metabolic remodeling, and repair-associated processes [35]. These findings indicate that M1-like and M2-like labels are best regarded as descriptive terms for functional tendencies rather than as fixed microglial subtypes with discrete boundaries.

Single-cell transcriptomic analyses have begun to address the limitations of the traditional M1/M2 framework and have advanced the study of macrophage and microglial activation toward more functionally informative transcriptional axes. CXCL9 and SPP1 have been used to define relatively distinct functional programs in myeloid cells: CXCL9 is associated with pro-inflammatory responses, whereas SPP1 is linked to repair- and tissue-remodeling-related responses [40]. This classification framework has also been extended to the central nervous system. In cross-disease myeloid cell atlases covering multiple human and mouse disease conditions, SPP1-associated cell populations substantially overlap with phagocytic and repair-related functions [41]. In tumor-related studies, SPP1-positive myeloid cells are often associated with hypoxia-related microenvironments and tissue remodeling programs [40]. This SPP1-associated repair program partially overlaps with the immunoregulatory, phagocytic, and tissue-remodeling features attributed to M2d-like myeloid responses, although the two frameworks should not be considered equivalent. This transcriptional axis provides a perspective beyond the traditional M1/M2 framework for studying vascular repair after stroke and may help clarify dynamic relationships among inflammation, hypoxic adaptation, phagocytic clearance, and tissue remodeling.

**Table 2:** Conventional M1-like and M2-like functional programs and their proposed relationships with post-stroke vascular repair.

Functional Program	Inducing Factors or Stimuli	Common Markers	Associated Mediators	Major Functions	Proposed Relevance to Post-Stroke Vascular Repair	Ref.
M1-like	DAMPs and TLR ligands, IFN-γ, TNF-α, ischemia-related ROS	iNOS, CD86, CD16 or CD32, MHC II	TNF-α, IL-1β, IL-6, NO and ROS	Pro-inflammatory response, damage recognition, immune-cell recruitment, and secondary injury	Early activation may contribute to damage recognition and debris clearance. Persistent activation may amplify endothelial inflammation, disrupt blood–brain barrier integrity, and impair neurovascular unit repair.	[27,32,33,35]
M2a-like	IL-4, IL-13, M-CSF	CD206, Arg1, CD209	IL-10, TGF-β, IGF-1	Anti-inflammatory response, phagocytosis, tissue repair	May contribute to an anti-inflammatory and tissue-repair microenvironment, thereby indirectly supporting vascular repair through inflammation resolution, phagocytic clearance, and trophic factor production.	[36,42]
M2b-like	Immune complexes with TLR or IL-1R ligands, LPS, IL-1β	CCL1, IL-10, CD86	IL-10, TNF-α, IL-6	Immunoregulation, inflammatory balance, mixed inflammatory response	May help buffer excessive inflammatory responses around injured vessels.	[36,43]
M2c-like	IL-10, TGF-β, glucocorticoids, M-CSF	CD163, MerTK, CD206	IL-10, TGF-β, MMPs	Efferocytosis, inflammation resolution, matrix remodeling	May favor vascular repair by promoting apoptotic or necrotic cell clearance, resolving local inflammation, and remodeling the extracellular matrix.	[36,44,45]
M2d-like	Adenosine and A2A receptor signaling with TLR ligands, IL-6	CD163, A2A receptor-associated phenotype	VEGF-A, IL-10, G-CSF	Pro-angiogenic response, immunoregulation, tissue remodeling	Potentially links immunoregulation with angiogenic signaling; direct evidence in post-stroke microglia remains limited.	[28,46,47]

Note: M1-like, M2-like, and M2d-like designations represent approximate and potentially overlapping functional programs rather than fixed, mutually exclusive cellular categories.

### 3.2. M2d-like Features in Repair-Associated Microglial Responses and Vascular Network Remodeling After Ischemic Stroke

The M2d-like program has been defined primarily in studies of tumor-associated macrophages, whereas direct evidence in post-stroke microglia remains limited. In tumor-associated macrophage studies, the M2d-like program is typically associated with high IL-10 expression, low IL-12 expression, CD163 upregulation, and angiogenesis- and immunoregulation-related secretory features [46]. In tumor and chronic inflammatory microenvironments, the M2d phenotype can be induced by IL-6, LIF, adenosine receptor-related signaling, and Toll-like receptor-associated stimulation (Table 3). These responses are associated with angiogenesis, immunoregulation, and tissue remodeling [46,48]. Thus, the conceptual value of the M2d-like framework lies in linking immunoregulation with angiogenesis-related programs. This conceptual framework may help interpret the pro-vascular functions of repair-associated microglia after stroke.

After ischemic stroke, the proposed contribution of microglial responses with M2d-like features to vascular network remodeling is supported mainly by evidence from repair-associated microglia. VEGFA-positive repair-associated microglia have been identified in the human stroke penumbra and in experimental models of cerebrovascular injury. These cells emerge during vascular reconstruction, and their reparative programming is regulated by IL-6 signaling and contributes to the remodeling of injured cerebral vessels [51]. IL-6 deficiency or impaired IL-6Rα signaling in microglia reduces the generation of repair-associated microglia. Impaired microglial IL-6 signaling is accompanied by reduced vascular coverage, compromised blood–brain barrier integrity, neuronal loss, and limited functional recovery [51]. These findings indicate that microglia can acquire pro-vascular reparative features after cerebrovascular injury that functionally overlap with the angiogenic component of the classical M2d phenotype.

**Figure 2:**
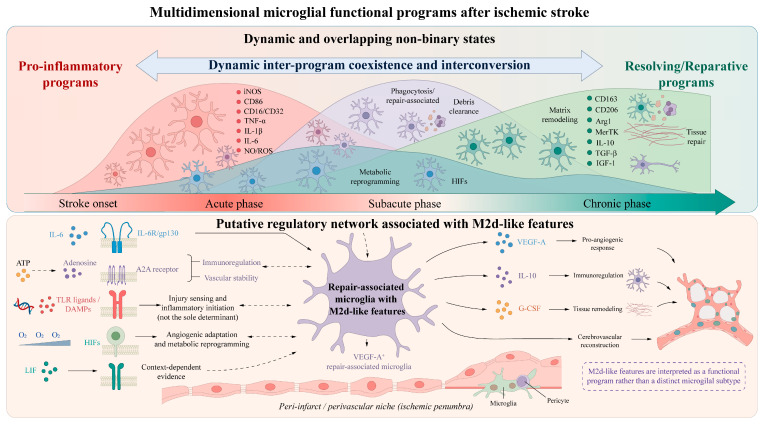
Dynamic microglial functional programs and putative regulation of M2d-like features after ischemic stroke. Microglia exhibit high plasticity and adopt overlapping transcriptional, metabolic, inflammatory, phagocytic, and reparative programs rather than transitioning along a fixed M1/M2 binary axis. At stroke onset and during the acute phase, damage-associated signals predominantly promote pro-inflammatory programs accompanied by metabolic stress responses, with increased expression of iNOS, CD86, TNF-α, and IL-1β that may contribute to secondary injury and blood–brain barrier disruption. As injury progresses into the subacute and chronic phases, microglial responses increasingly involve debris clearance, matrix remodeling, and tissue repair, with expression of markers such as CD163, CD206, Arg1, and MerTK. Within the peri-infarct and perivascular niches, IL-6, adenosine/A2A receptor signaling, TLR-related injury signals, HIF-mediated hypoxic responses, and LIF may collectively contribute to a candidate repair-associated microglial program with M2d-like features and increased VEGF-A expression. Repair-associated microglia with M2d-like features may subsequently release mediators such as VEGF-A, IL-10, and G-CSF, thereby supporting angiogenic signaling, immunoregulation, tissue remodeling, and stabilization of the perivascular environment, and potentially contributing to vascular repair and neurological recovery after ischemic stroke. The figure was created using Adobe Illustrator 2024 (Adobe Inc., San Jose, CA, USA).

In the post-ischemic penumbra, the functional state of microglia is not determined by a single stimulus. Instead, microglial responses are shaped by the combined demands of injury recognition, inflammatory regulation, hypoxic adaptation, and vascular repair. IL-6 is currently the signal most directly linked to repair-associated microglial programming after cerebrovascular injury. Monocyte-derived IL-6 promotes the acquisition of proliferative and pro-angiogenic properties by VEGFA-positive repair-associated microglia, thereby linking infiltrating peripheral monocytes to cerebrovascular repair [50,51]. As extracellular ATP is converted to adenosine, A2A receptor signaling may contribute to post-ischemic inflammatory regulation and support pericyte and blood–brain barrier stability, thereby creating a vascular environment favorable for repair [53,54]. TLR-related signaling contributes to injury recognition and inflammatory activation, whereas HIF-mediated responses may support adaptation to the hypoxic microenvironment [55,56]. In contrast, evidence linking LIF to M2d-like differentiation is derived mainly from tumor-associated monocyte/macrophage models [46,48]. These factors should not be interpreted as independent activators acting in isolation. Rather, they may constitute an interconnected regulatory framework linking injury recognition, inflammatory regulation, hypoxic adaptation, and vascular repair (Figure 2).

Overall, the M2d-like framework provides a useful perspective for understanding the link between inflammatory regulation and vascular network remodeling after stroke. The coexistence of pro-angiogenic and immunoregulatory features within this framework may help explain the transition of the penumbral microenvironment from injury-dominated responses toward tissue repair. Future studies integrating single-cell omics, spatial transcriptomics, lineage tracing, and functional vascular assessment are needed to determine whether microglial populations with M2d-like features arise in the penumbra and contribute to vascular repair.

## 4. Putative Mechanisms Linking Repair-Associated Microglial Responses with M2d-like Features to Vascular Network Remodeling

**Figure 3:**
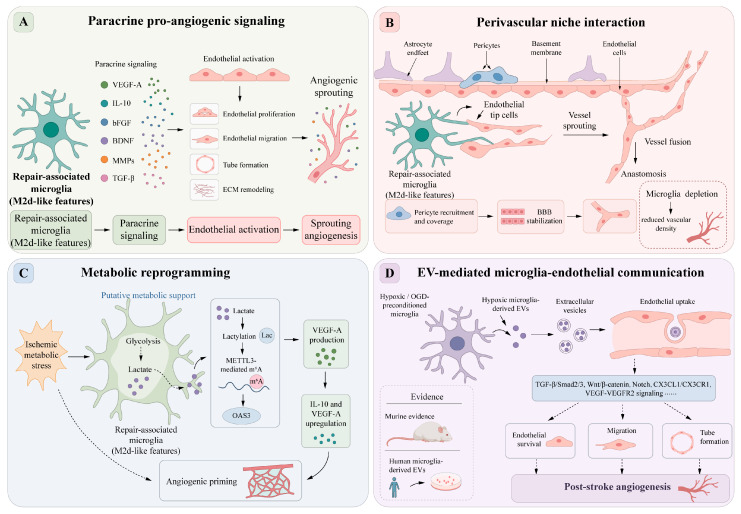
Putative mechanistic layers linking repair-associated microglial responses with M2d-like features to vascular network remodeling after ischemic stroke. (**A**). **Paracrine pro-angiogenic signaling.** Repair-associated microglia with M2d-like features may release soluble mediators, including VEGF-A, IL-10, bFGF, BDNF, MMPs, and TGF-β. These mediators may collectively support endothelial activation, proliferation, migration, tube formation, and extracellular matrix (ECM) remodeling, thereby promoting angiogenic sprouting. (**B**). **Perivascular niche interaction.** Within the neurovascular unit, repair-associated microglia may interact with endothelial tip cells, pericytes, and the basement membrane during vessel sprouting, fusion, and anastomosis. Microglia-associated pericyte recruitment and coverage may contribute to blood–brain barrier (BBB) stabilization, whereas microglial depletion has been associated with reduced vascular density. (**C**). **Metabolic reprogramming.** Ischemic metabolic stress may promote glycolysis and lactate accumulation in repair-associated microglia. Based mainly on cross-model evidence, lactate-related signaling may involve protein lactylation and METTL3-mediated m6A regulation of OAS3, potentially contributing to VEGF-A- and IL-10-associated reparative outputs. This proposed pathway requires validation in post-stroke microglia. (**D**). **EV-mediated microglia–endothelial communication.** Hypoxic or oxygen–glucose deprivation (OGD)-preconditioned microglia release extracellular vesicles (EVs) that can be internalized by endothelial cells. Evidence from murine models and human microglia-derived EV systems suggests that these EVs may activate downstream pathways, including TGF-β/Smad2/3, Wnt/β-catenin, Notch, CX3CL1/CX3CR1, and VEGF-VEGFR2 signaling, thereby supporting endothelial survival, migration, tube formation, and post-stroke angiogenesis. The figure was created using Adobe Illustrator 2024 (Adobe Inc., San Jose, CA, USA).

Current evidence indicates that the pro-vascular effects of repair-associated microglial responses cannot be attributed to a single mediator or signaling pathway. Rather, these effects may emerge from coordinated immunoregulatory, metabolic, spatial, and intercellular communication programs shaped by the ischemic microenvironment. Stroke-related evidence further shows that inflammatory cues can induce repair-associated microglial programs involved in the reconstruction of damaged cerebral vessels [51]. Accordingly, the potential contribution of microglial responses with M2d-like features to post-stroke vascular remodeling is examined through four interrelated mechanistic dimensions: paracrine signaling, perivascular interactions, metabolic reprogramming, and extracellular vesicle-mediated communication (Figure 3).

### 4.1. Paracrine Pro-Angiogenic Factors

After ischemic stroke, repair-phase microglia may modulate endothelial behavior and vascular repair in the penumbra through soluble mediators. Rather than acting through a single angiogenic factor, microglia-associated paracrine responses are likely to involve coordinated angiogenic, matrix-remodeling, and immunoregulatory signals. These signals may collectively support endothelial activation, vascular extension, and stabilization of the local repair milieu [57]. This paracrine network provides a plausible basis for linking repair-associated microglial responses with M2d-like features to vascular network remodeling. However, whether repair-associated microglia acquire a reproducible secretory profile with M2d-like features in the ischemic brain, and the extent to which such responses contribute to vascular repair, remain to be determined.

Among these mediators, VEGF-A is among the most strongly supported molecules implicated in microglia-associated vascular repair. In the stroke penumbra and in models of cerebrovascular injury, VEGFA-positive repair-associated microglia have been associated with vascular reconstruction, providing disease-relevant evidence that microglia may contribute to post-stroke cerebrovascular repair [51]. IL-10-mediated immunoregulation may attenuate secondary inflammatory injury affecting the vascular wall and blood–brain barrier, thereby maintaining a local environment permissive for endothelial repair [58]. Classical M2d myeloid cells are commonly characterized by VEGF- and IL-10-related angiogenic and immunoregulatory features. Therefore, the emergence of VEGFA-positive microglia after stroke shows functional overlap with key features of classical M2d myeloid cells, although these populations should not currently be regarded as equivalent [59].

Additional paracrine mediators may further contribute to vascular structural remodeling and stabilization of the neurovascular unit. Basic fibroblast growth factor may cooperate with VEGF-related pathways to support endothelial proliferation and migration, whereas matrix metalloproteinases regulate extracellular matrix degradation and remodeling during vascular extension and tissue reconstruction. Brain-derived neurotrophic factor is more closely linked to neuronal survival, synaptic repair, and preservation of neurovascular unit function [57,60,61]. TGF-β may also participate in the repair microenvironment through immunoregulatory and matrix-remodeling activities; however, its role in vascular repair is likely context- and timing-dependent rather than uniformly pro-angiogenic. Together, these signals may support remodeling of the penumbral microenvironment and correspond to the paracrine layer illustrated in Figure 3A. At present, the evidence most directly supports a role for VEGFA-positive repair-associated microglia in cerebrovascular repair, whereas the full paracrine signature of repair-associated microglia with M2d-like features after stroke remains unclear.

### 4.2. Cellular Interactions Within the Perivascular Microenvironment

The perivascular microenvironment provides a critical anatomical and functional interface for interactions between microglia and vascular cells within the neurovascular unit. Brain microvascular endothelial cells, pericytes, basement membrane components, astrocytic endfeet, and microglia collectively form a local regulatory niche that integrates immune and vascular signals. Microglial processes can contact endothelial cells, pericytes, vascular smooth muscle cells, and astrocytes, thereby contributing to the regulation of local blood flow, vascular tone, and blood–brain barrier permeability. During angiogenesis in the central nervous system, microglia can associate with endothelial tip cells and contribute to vascular sprout fusion and network formation [62,63]. These observations indicate that microglia are spatially positioned to influence vascular remodeling within the perivascular niche.

Ischemic stroke converts the perivascular niche from a homeostatic interface into a site of injury response and reparative remodeling. In the penumbra, blood–brain barrier disruption, endothelial activation, inflammation, and microcirculatory dysfunction occur concurrently; therefore, restoration of the vascular network requires coordinated interactions between immune and vascular cells. In a mouse model of transient middle cerebral artery occlusion, targeting inflamed vascular endothelium and releasing H_2_ at the blood–brain barrier interface improved barrier integrity and cerebral perfusion, promoted revascularization in infarct and peri-infarct regions, and coincided with a shift in microglia from a pro-inflammatory state toward an ARG1-associated reparative state. In contrast, microglial depletion markedly impaired vascular ingrowth and axonal regeneration [64]. These findings suggest that post-stroke vascular network repair depends not only on endothelial responses but also on the functional state of microglia within the perivascular environment, as summarized in Figure 3B.

Evidence directly linking repair-associated microglial responses with M2d-like features to perivascular remodeling after ischemic stroke remains limited. Most related evidence is derived from glioblastoma and other myeloid cell models, in which glioma-associated microglia or macrophages with M2d-like pro-vascular and immunoregulatory features have been observed within perivascular niches. These cells may produce factors such as VEGF-A, IL-10, TGF-β, MMP-2, and MMP-9. VEGF-A is associated with angiogenic signaling, MMP-2 and MMP-9 contribute to extracellular matrix degradation and perivascular matrix remodeling, whereas IL-10 and TGF-β are consistent with an immunoregulatory secretory profile [65,66]. Although these findings provide a useful conceptual framework for understanding how M2d-like myeloid cells may contribute to vascular repair, they should be interpreted cautiously when applied to stroke-specific microglia. Thus, the perivascular microenvironment may provide a spatial context for repair-associated microglial responses with M2d-like features during post-stroke vascular remodeling; however, their precise localization, inducing conditions, and functional contribution require validation in ischemic stroke models.

### 4.3. Metabolic Reprogramming and the Initiation of Pro-Vascular Functions

Metabolic reprogramming is a central mechanism by which myeloid cells adapt their functional states to injured microenvironments. This process is not limited to glycolysis or lactate production; instead, it involves coordinated changes in glucose metabolism, lipid metabolism, amino acid metabolism, the tricarboxylic acid cycle, and oxidative phosphorylation [67,68]. These metabolic pathways shape the inflammatory, reparative, matrix-remodeling, and pro-vascular functions of myeloid cells. Integrated metabolomic and transcriptomic analyses suggest that M2d macrophages may display metabolic and functional features distinct from other M2-like states and associated with angiogenesis and extracellular matrix organization [69]. This profile is consistent with a repair-associated secretory program involving IL-10, TGF-β, and VEGF-related signals [68]. Therefore, the pro-vascular activity associated with repair-associated myeloid cells exhibiting M2d-like features is better understood as an outcome of coordinated metabolic and secretory remodeling rather than the isolated consequence of a single angiogenic mediator.

Within this metabolic network, lactate may serve as a signaling node that links the ischemic metabolic environment to repair-associated secretory programs. After cerebral ischemia, lactate accumulates in ischemic brain tissue, accompanied by increased protein lactylation in the penumbra. Inhibition of glycolysis or lactate transport reduces lactylation and aggravates brain injury, whereas lactate supplementation improves neurological outcomes after ischemia [70]. Evidence from myeloid cells further indicates that lactate-related signaling may promote an M2d-like phenotype characterized by high IL-10 and VEGF-A expression, potentially through METTL3-m6A-OAS3-related regulatory mechanisms [71]. Studies of peripheral tissue repair also show that pro-healing responses are often accompanied by increased M2 macrophage abundance, elevated VEGFA expression, and enhanced angiogenesis, with M2d-associated phenotypes appearing more prominently during the middle-to-late stages after injury [72,73]. These findings suggest that lactate-related metabolic signaling may contribute to the establishment of reparative secretory programs, as illustrated in Figure 3C. However, because much of this evidence is derived from macrophage studies and cross-model analyses, it should not be directly equated with M2d-like microglial responses in the stroke penumbra.

Stroke-related microglial studies provide an intracerebral context in which these mechanisms can be interpreted. After acute ischemia, microglia exhibit spatially dependent metabolic heterogeneity. Microglia associated with the ischemic core tend to show stronger glycolytic and inflammatory features, whereas those in the penumbra are more closely associated with tricarboxylic acid cycle activity and oxidative phosphorylation, a metabolic profile linked to a relatively attenuated inflammatory response [74]. Vascular-associated microglia can also adopt a metabolically active state characterized by enhanced glycolysis and oxidative phosphorylation; these programs are associated with blood–brain barrier disruption and vascular regeneration outcomes [75]. Taken together, current evidence suggests that metabolic remodeling within the ischemic penumbra may create a permissive microenvironment for repair-associated microglial functions. Nevertheless, whether repair-associated microglia with M2d-like features contribute directly to post-stroke vascular repair through metabolic remodeling remains unresolved.

### 4.4. Extracellular Vesicle-Mediated Communication Between Microglia and Endothelial Cells

Extracellular vesicles are key mediators of intercellular communication in the central nervous system. They carry bioactive cargo, including proteins, lipids, and nucleic acids, and their composition is influenced by both the state of the parent cell and external stimuli [76]. In ischemic stroke, microglia communicate with neurons, astrocytes, endothelial cells, and peripheral immune cells through multiple signaling routes. Alongside soluble mediators and direct cell–cell contact, extracellular vesicles participate in inflammatory regulation, tissue repair, and the maintenance of blood–brain barrier homeostasis [77]. Thus, extracellular vesicle-mediated signaling represents a contact-independent route through which microglia may influence the vascular unit.

Ischemic or hypoxic stimulation may alter the functional properties of microglia-derived extracellular vesicles, enabling them to regulate endothelial behavior. Extracellular vesicles derived from oxygen–glucose deprivation-preconditioned microglia can be taken up by endothelial cells and promote endothelial survival, migration, and tube formation. In mouse models of stroke, these vesicles also enhance angiogenesis-related responses and improve neurological recovery [78]. Similarly, extracellular vesicles derived from hypoxia-preconditioned human microglia enhance the migration and tube formation of brain microvascular endothelial cells. In zebrafish and mouse models of middle cerebral artery occlusion, these vesicles promote vascular formation or increase microvascular density in peri-ischemic regions [79]. These findings indicate that microglia-derived extracellular vesicles may transmit injury-related signals from the ischemic microenvironment to endothelial cells and thereby contribute to vascular network remodeling after stroke, as shown in Figure 3D.

Extracellular vesicle-mediated communication differs from local paracrine signaling because it involves packaged, transferable molecular information rather than freely soluble mediators. Current studies support the involvement of extracellular vesicles derived from ischemia- or hypoxia-conditioned microglia in endothelial regulation and post-stroke vascular repair. However, the relationship between pro-reparative extracellular vesicles and repair-associated microglial responses with M2d-like features remains unresolved. For repair-associated microglia with M2d-like features, the extracellular vesicle cargo profile, endothelial-targeting capacity, and functional contribution to vascular remodeling within the penumbra require further investigation through cell-of-origin tracing, cargo manipulation, and endothelial uptake experiments.

Overall, repair-associated microglial responses with M2d-like features should be considered within a working framework that integrates several repair-related mechanisms, rather than as a fully established stroke-specific microglial subtype. Within this framework, the perivascular microenvironment provides a spatial context for sensing vascular injury; paracrine mediators shape endothelial and matrix responses; metabolic remodeling supports repair-associated functional states; and extracellular vesicles extend communication between microglia and endothelial cells. Together, these mechanisms outline a putative immunovascular repair axis after ischemic stroke. The key challenge for future studies is to determine whether this axis is mediated by a distinct M2d-like microglial population or by broader repair-associated microglial programs exhibiting M2d-like features.

## 5. Therapeutic Implications of Repair-Associated Microglial Responses with M2d-like Features in Post-Stroke Vascular Repair

### 5.1. Therapeutic Plasticity of Repair-Associated Microglial Responses

Based on the temporal heterogeneity of microglia described above, post-stroke microglial responses should be viewed not as fixed states but as processes with pharmacological plasticity. Minocycline can reduce M1-like responses and promote M2-like polarization through regulation of STAT1/STAT6 signaling, suggesting that pro-inflammatory microglial responses after ischemia may be reversible [80]. Tranilast reduces inflammatory factor release by inhibiting the NLRP3 inflammasome and increases the expression of M2-like markers, further supporting the role of inflammasome signaling in reshaping repair-associated microglial responses [81]. Together, these findings highlight the therapeutic plasticity of post-stroke microglial responses. However, their interpretation remains largely limited to broadly defined repair-associated phenotypes and cannot yet be directly attributed to an M2d-like cellular state.

In the context of stroke, discussion of repair-associated microglial responses with M2d-like features should focus on the functional link between immunoregulation and vascular repair. Studies of peripheral macrophages suggest that the M2d state is associated with IL-10-mediated immunoregulation and VEGF-A-related vascular signaling and may therefore provide a functional reference for defining repair-associated microglial states after stroke [66]. Evidence that the Fra-1/IL-6 axis contributes to M2d macrophage generation in tumor co-culture systems further indicates that the M2d state is highly microenvironment-dependent [82]. Given these evidentiary limitations, M2d-like features in post-stroke microglial responses are better viewed as an analytical framework for assessing whether reparative immune responses extend to vascular repair, rather than as evidence of a predefined and independent microglial subtype.

### 5.2. Evidence from Traditional Chinese Medicine on Immune–Vascular Coupled Repair

The reparative significance of post-ischemic microglial responses should be evaluated by considering both immunoregulatory signals and vascular repair outcomes, rather than relying solely on increased expression of M2-like markers. Studies of active compounds, single-herb extracts, and traditional Chinese medicine formulas suggest that immunomodulation can occur in parallel with vascular repair. Interventions using compounds such as astragaloside IV and berberine indicate that regulation of metabolic inflammation may be linked to VEGF-A, BDNF, IGF-1, or endothelial angiogenic responses [83,84]. Albiflorin, another natural monoterpene glycoside, has been reported to exert anti-inflammatory, antioxidant, and neuroprotective effects in experimental models of cerebral ischemia, although its relevance to microglial identity and vascular remodeling remains unclear [85]. Building on this evidence, studies of single-herb interventions such as Gastrodia elata Blume and earthworm extract further suggest that suppression of inflammatory signaling may be accompanied by enhanced reparative outputs, including IL-10, TGF-β, VEGF-A, and Ang1/Tie2/Ang2 signaling [86,87]. Compared with isolated compounds and single-herb interventions, formulas such as Yi Qi Huo Xue Fang and Buyang Huanwu Decoction show broader regulatory effects. These formulas can modulate microglial polarization, endothelial responses, cerebral blood flow recovery, and neurovascular remodeling [88,89]. The broader literature on marine traditional Chinese medicines similarly illustrates the multi-component and multi-target nature of natural-product interventions, with reported anti-inflammatory, antioxidant, immunoregulatory, and angiogenesis-related activities in diverse non-stroke models [90]. Collectively, these findings suggest that traditional Chinese medicine interventions may influence inflammatory responses and vascular repair-related processes in parallel. However, the cellular sources involved and causal relationships underlying these functional effects require further validation.

The association between traditional Chinese medicine research and repair-associated microglial responses with M2d-like features should be restricted to the functional level of concurrent immunoregulation and pro-vascular repair. Current evidence has not yet simultaneously covered features more closely aligned with repair-associated microglial responses with M2d-like features, such as CD163, A2A receptor, IL-10, and VEGF-A. In addition, preparation standardization and bioavailability are often insufficiently reported, while validation of microglial origin, perivascular localization, and long-term vascular outcomes remains lacking. Therefore, evidence from active compounds, single-herb interventions, and traditional Chinese medicine formulas is more appropriate for supporting the possibility that repair-associated microglial responses contribute to vascular remodeling than for directly concluding that a distinct M2d-like microglial population has been specifically induced (Table 4).

### 5.3. The Value of EV- and Nanodelivery-Based Systems for Spatiotemporal Regulation

Unlike Chinese herbal monomers and formulas, which primarily regulate the inflammatory vascular microenvironment, EV- and nanodelivery-based systems enable targeted delivery and timed release of repair signals at lesion sites. Exosome-related pathways associated with Buyang Huanwu Decoction may promote angiogenesis through the MALAT1/YAP1/HIF-1α axis, suggesting that herbal formulas may influence vascular repair through intercellular signal transfer [92]. Building on this evidence, Houshiheisan-modified EPC-derived EVs can enhance endothelial cell proliferation, migration, and tube formation through the miR-126/PIK3R2/PI3K/AKT axis, indicating that EVs may serve as delivery platforms for pro-vascular repair signals [93]. Plant-derived EVs from Chuanxiong further suggest that herb-derived vesicles may combine brain-targeted delivery with neurovascular repair potential [94]. These findings indicate that the main value of EVs lies in regulating intercellular communication and repair-signal transmission after stroke, rather than directly demonstrating the induction of M2d-like microglia.

Nanodelivery systems further emphasize lesion-responsive release and spatially precise regulation. ROS-responsive sulfated polysaccharide nanocarriers can release active compounds in the highly oxidative ischemic microenvironment while modulating microglial polarization, preserving blood–brain barrier integrity, and promoting neurovascular remodeling [95]. Rutin-based bimetallic phenolic nanoparticles can alleviate oxidative stress, modulate microglial polarization, and promote vascular normalization [96]. Compared with conventional drug administration, these systems may be better suited for reparative immune regulation within the penumbra and perivascular niches. Despite these encouraging preclinical findings, their clinical translation remains limited by insufficient evidence regarding optimal dosing, treatment timing, delivery routes, long-term safety, large-scale manufacturing, and validation in human studies. For plant-derived EVs, controllable culture platforms such as temporary immersion bioreactor systems may help improve batch consistency, production standardization, and scalability [97]. Future investigations integrating cellular identity, spatial localization, and functional vascular outcomes will therefore be essential before these therapeutic strategies can be translated into clinical practice.

## 6. Challenges and Future Directions for Validation

Whether M2d-like microglia after stroke represent a distinct repair-associated state remains to be defined through rigorous cellular identification. The concept of M2d polarization was mainly derived from studies of peripheral macrophages, tumor-associated macrophages, and mucosal repair and is typically characterized by IL-10-mediated immune regulation, VEGF-A-associated pro-angiogenic activity, and low IL-12 expression [66,82]. However, microglia in the ischemic penumbra are simultaneously influenced by hypoxia, amplified inflammation, metabolic stress, cellular debris clearance, and vascular repair signals. Their state may therefore not be equivalent to that of peripheral M2d macrophages. Moreover, markers such as Arg1, CD206, and IL-10 can appear in myeloid cells from different tissues and under different stimuli. When used alone, these markers may cause reparative tendencies to be misinterpreted as specific activation states [98]. Consistent with single-cell evidence showing spatial and temporal heterogeneity of mouse and human microglia [99], future studies should integrate single-cell transcriptomics, spatial transcriptomics, lineage tracing, microglia-specific marker panels, and perivascular spatial mapping to determine whether the post-stroke penumbra contains microglial populations with both immunoregulatory and pro-vascular repair features.

Even if M2d-like microglia can be identified, their therapeutic relevance depends on whether they contribute to functional vascular remodeling. Post-stroke vascular repair should not be evaluated solely by increases in VEGF, CD31, or microvessel density. If newly formed vessels lack mature lumens, effective perfusion, pericyte coverage, and blood–brain barrier stability, their capacity to support long-term neurological recovery remains limited [33]. Therefore, future studies should combine structural angiogenic measures with functionally informative endpoints, including pericyte coverage, vascular leakage or tracer extravasation, longitudinal perfusion imaging, serial neurological scores, white matter repair, and spatial mapping of lineage-defined microglia relative to remodeling vessels [33]. In studies of traditional Chinese medicine, whether an intervention truly regulates an M2d-like reparative state can be determined only when microglial origin is clearly defined, spatial localization is established, and vascular repair endpoints are functionally validated.

In addition, translational studies of traditional Chinese medicine should be aligned with the clinical course of stroke and supported by human-derived evidence. Studies in patients with acute ischemic stroke have shown that pericyte-derived microvesicles increase in a time-dependent manner after stroke, with their cargo gradually shifting from early anti-inflammatory signals toward pro-angiogenic and vascular remodeling-related signals [100]. This dynamic change suggests that post-stroke repair involves measurable time windows and microvascular signaling features. Accordingly, studies of traditional Chinese medicine should distinguish between pathological features of the acute and subacute phases and separately evaluate inflammation control, barrier protection, immune regulation, vascular maturation, and perfusion recovery. Spatial metabolomics may provide a complementary approach for mapping the tissue distribution and temporal transformation of bioactive components in traditional Chinese medicine interventions, although its application to post-stroke neurovascular repair remains to be established [101]. Integrating human-derived microglial models, clinical biomarker data, and long-term functional outcomes will improve the interpretability and translational value of traditional Chinese medicine in regulating reparative immunovascular responses.

## 7. Conclusions

The concept of repair-associated microglial responses with M2d-like features provides a candidate framework for understanding the link between immunoregulation and vascular repair after ischemic stroke. Compared with conventional M2-like polarization, the conceptual value of the proposed M2d-like state lies in emphasizing the coexistence of immunoregulatory activity and pro-vascular repair outputs, rather than simply describing an increase in anti-inflammatory markers.

Chinese herbal monomers, herbal formulas, EVs, and nanodelivery systems provide complementary therapeutic strategies for exploring repair-associated immunovascular responses, although current evidence does not demonstrate that these interventions specifically induce M2d-like microglial states. Herbal monomers and formulas mainly support the parallel regulation of immune responses and vascular repair, whereas EV- and nanodelivery-based systems may improve the targeted delivery and spatial control of repair signals. Future studies should integrate single-cell transcriptomics, spatial mapping, lineage tracing, and comprehensive vascular outcome assessments to clarify the cellular identity, spatial localization, and functional contribution of repair-associated microglia before these therapeutic strategies can be translated into clinical practice.

## Figures and Tables

**Table 1:** Developmental origin and post-stroke accumulation of resident microglia and peripheral myeloid cells.

Cell Population	Source or Process	Biological Relevance	Lineage Markers and Identification Approaches	Notes and Interpretive Limitations	Ref.
Resident microglia	Yolk sac-derived progenitors	Establishment of the pre-existing CNS-resident microglial population	TMEM119, P2RY12, and SALL1 used in combination	Developmental origin rather than a newly recruited source after stroke; no single marker is definitive	[23,24,27]
	Local proliferation and self-renewal	Expansion of pre-existing resident microglia after CNS injury	Resident microglial markers combined with proliferation or repopulation evidence	Expansion of an existing lineage; evidence includes CNS injury, depletion, and homeostatic models and is not uniformly stroke-specific	[25,26,29]
	Redistribution from adjacent regions	May contribute to local accumulation around injured tissue	Resident microglial markers combined with spatial assessment	Mainly supported by the broader CNS injury literature; direct lineage-resolved evidence after ischemic stroke remains limited	[25]
Peripheral myeloid cells	Circulating monocytes/monocyte-derived macrophages	Recruitment into ischemic tissue and participation in inflammatory and repair responses	CD11b^high/CD45^high; CCR2-based recruitment analysis; Ly6C/CX3CR1 subset profiling	Applied in a mouse MCAO model; these cells remain developmentally distinct from resident microglia, and marker combinations are required for lineage interpretation	[21,27,30]
	Bone marrow-derived cells under manipulated conditions	Peripheral-cell contribution after irradiation, transplantation, or experimental microglial depletion	Donor-cell labeling and bone marrow chimerism	Experimental preconditioning may alter BBB integrity and increase peripheral-cell entry; findings should not be generalized to unmanipulated stroke	[25,26]

**Table 3:** Candidate signals and mechanisms relevant to repair-associated microglial responses with M2d-like features and post-stroke vascular remodeling.

Activator	Mechanism of Action	Functional Outcomes	Evidence Level	Ref.
IL-6	1. Activates IL-6R- and gp130-related JAK/STAT signaling. 2. Promotes repair-associated microglial programming under injury conditions. 3. Enhances angiogenesis-related and immunoregulatory responses.	1. Enhances VEGFA-positive repair-associated microglial responses. 2. Supports cerebrovascular reconstruction. 3. Contributes to neurological recovery after vascular injury.	Direct evidence for repair-associated microglial programming and cerebrovascular repair; M2d-like identity remains inferential.	[49,50,51,52]
Adenosine A2A receptor signaling	1. Binds to adenosine A2A receptors. 2. Activates A2A receptor-mediated immunoregulatory responses. 3. May provide permissive cues for M2d-like polarization under ischemic or inflammatory conditions.	1. Supports the vascular repair microenvironment. 2. Contributes to blood–brain barrier and perivascular cell stability. 3. Mitigates excessive inflammation after ischemic injury.	Direct evidence for A2A receptor-mediated pericyte protection in cerebral hypoperfusion; relevance to M2d-like microglia remains inferential.	[53,54]
TLR ligands with adenosine or IL-6	1. Activates TLR-related damage-recognition pathways. 2. Initiates microglial activation after ischemic injury. 3. May cooperate with adenosine- or IL-6-related signals during M2d-like phenotypic transition.	1. Supports inflammatory adjustment after tissue injury. 2. Promotes repair-associated immune modulation. 3. Facilitates tissue remodeling under appropriate repair conditions.	Direct evidence for TLR-mediated microglial activation after stroke; M2d-like co-stimulation is supported by macrophage-based studies.	[55,56]
HIFs	1. Are stabilized under hypoxic conditions. 2. Enhance hypoxia-adaptive responses in ischemic or low-oxygen microenvironments. 3. Support angiogenesis-related transcriptional activity.	1. Supports vascular network formation. 2. Promotes tissue adaptation to low-oxygen stress. 3. Provides a permissive background for angiogenic remodeling.	M2d-related evidence derives from tumor-associated macrophage and computational studies; post-stroke microglial validation is lacking.	[46,56]
LIF	1. Induces IL-6- or M-CSF-associated autocrine regulation. 2. Skews monocyte-derived macrophages toward M2d-like phenotypes. 3. Supports immunoregulatory differentiation rather than dendritic-cell differentiation.	1. Supports immunoregulation. 2. Promotes pro-angiogenic remodeling. 3. Provides indirect support for M2d-like repair responses.	Direct evidence for TAM-like macrophage differentiation in tumor models; post-stroke microglial validation is lacking.	[46,48]

**Table 4:** Representative experimental evidence linking traditional Chinese medicine interventions to repair-associated microglial responses and vascular repair after ischemic stroke.

Traditional Chinese Medicine Intervention	Type	Experimental Model (Treatment Protocol)	Species	Main Mechanism	Microglial Markers/Effects	Vascular Repair-Related Effects	Relationship to M2d-like Features	Level of Evidence	Ref.
Astragaloside IV	Monomer	tMCAO; i.p.; 40 mg/kg/day; 14 d	Male SD rats	PPARγ	CD206, Arg1, IL-10 ↑; CD86, iNOS ↓	VEGF-A, BrdU^+^vWF^+^ cells ↑; angiogenesis ↑	Indirect support; no direct M2d-like validation	Direct experimental stroke evidence	[83]
Berberine	Monomer	tMCAO; gavage; 50 mg/kg/day; 14 d	Male C57BL/6 mice	AMPK	CD206, Arg1, IL-10 ↑; CD16/CD32, TNF-α ↓	CD31, CD31^+^Ki67^+^ cells ↑; angiogenesis ↑	Indirect support; no direct M2d-like validation	Direct mechanistic stroke evidence	[84]
Catalpol	Monomer	pMCAO; i.p.; 5 mg/kg/day; 7 d	Male SD rats	JAK2–STAT3–VEGF-A	Microglial phenotype not assessed	VEGF-A, vWF^+^PCNA^+^ vessels ↑; CBF ↑	Indirect support; no direct M2d-like validation	Direct mechanistic stroke evidence	[91]
Gastrodia elata Blume	Single-herb extract	tMCAO; i.p.; 0.5–1 g/kg; single dose	Male SD rats	JNK–TLR4–T3JAM–NF-κB	YM-1/2^+^Iba1^+^ cells ↑; CD86^+^Iba1^+^, TLR4^+^Iba1^+^ cells ↓	VEGF-A ↑; angiogenesis not directly assessed	Indirect support; no direct M2d-like validation	Direct mechanistic stroke evidence	[86]
Earthworm extract	Single-herb extract	tMCAO/R; i.v.; 1.6 mL/kg/day; 3 d	Male C57BL/6 mice	Ang1–Tie2–Ang2	CD206^+^Iba1^+^ cells, IL-10 ↑; CD16^+^Iba1^+^ cells, IL-1β, TNF-α ↓	VEGF, Ang1, Tie2, Ki67^+^LEL^+^ vessels ↑; Ang2 ↓	Indirect support; no direct M2d-like validation	Direct experimental stroke evidence	[87]
Yi Qi Huo Xue Fang	Herbal formula	tMCAO; gavage; 6.48 g/kg/day; days 2–14	Male SD rats	PPARG	CD206, Arg1, IL-10, TGF-β ↑; CD16, iNOS, TNF-α ↓	VEGFA, VEGFB, Ang1, CD31^+^Ki67^+^ cells ↑; vascular density ↑	Indirect support; no direct M2d-like validation	Direct experimental stroke evidence	[88]
Buyang Huanwu Decoction	Herbal formula	pMCAO; gavage; 16.6 g/kg/day; 30 d	Male SD rats	AMPK–CREB–NF-κB	ARG1^+^IBA1^+^ cells, CD206 ↑; CD16^+^IBA1^+^ cells, CD86, IBA1 ↓	VEGF, Ang1, CBF ↑; Ang2 ↓	Indirect support; no direct M2d-like validation	Direct experimental stroke evidence	[89]

## Data Availability

No new data were generated or analyzed in this review. Data sharing is not applicable to this article.

## Abbreviations

**A2A**	Adenosine A2A receptor
**AMPK**	AMP-activated protein kinase
**Ang1**	Angiopoietin-1
**Arg1**	Arginase-1
**BBB**	Blood–brain barrier
**BDNF**	Brain-derived neurotrophic factor
**bFGF**	Basic fibroblast growth factor
**CNS**	Central nervous system
**CX3CL1**	C-X3-C motif chemokine ligand 1
**CX3CR1**	C-X3-C motif chemokine receptor 1
**DAMPs**	Damage-associated molecular patterns
**ECM**	Extracellular matrix
**EPCs**	Endothelial progenitor cells
**EVs**	Extracellular vesicles
**G-CSF**	Granulocyte colony-stimulating factor
**HIFs**	Hypoxia-inducible factors
**HIF-1α**	Hypoxia-inducible factor 1 alpha
**IFN-γ**	Interferon gamma
**IGF-1**	Insulin-like growth factor 1
**IL**	Interleukin
**iNOS**	Inducible nitric oxide synthase
**JAK/STAT**	Janus kinase/signal transducer and activator of transcription
**JNK**	c-Jun N-terminal kinase
**LIF**	Leukemia inhibitory factor
**LPS**	Lipopolysaccharide
**MALAT1**	Metastasis-associated lung adenocarcinoma transcript 1
**MAPK/ERK**	Mitogen-activated protein kinase/extracellular signal-regulated kinase
**MCAO**	Middle cerebral artery occlusion
**M-CSF**	Macrophage colony-stimulating factor
**MerTK**	MER proto-oncogene tyrosine kinase
**METTL3**	Methyltransferase-like 3
**MHC II**	Major histocompatibility complex class II
**MMPs**	Matrix metalloproteinases
**m6A**	N6-methyladenosine
**NF-κB**	Nuclear factor kappa B
**NLRP3**	NLR family pyrin domain-containing 3
**NO**	Nitric oxide
**OAS3**	2′-5′-Oligoadenylate synthetase 3
**OGD**	Oxygen–glucose deprivation
**PDGF**	Platelet-derived growth factor
**PI3K/Akt**	Phosphoinositide 3-kinase/protein kinase B
**PIK3R2**	Phosphoinositide-3-kinase regulatory subunit 2
**PPARγ**	Peroxisome proliferator-activated receptor gamma
**ROS**	Reactive oxygen species
**SPP1**	Secreted phosphoprotein 1
**TAM**	Tumor-associated macrophage
**TGF-β**	Transforming growth factor beta
**Tie2**	Tyrosine kinase with immunoglobulin-like and EGF-like domains 2
**TLR**	Toll-like receptor
**TNF-α**	Tumor necrosis factor alpha
**VEGF-A**	Vascular endothelial growth factor A
**VEGFR2**	Vascular endothelial growth factor receptor 2
**YAP1**	Yes-associated protein 1
